# Preparation, Characterization and In Vitro Biological Evaluation of a Novel Pearl Powder/Poly-Amino Acid Composite as a Potential Substitute for Bone Repair and Reconstruction

**DOI:** 10.3390/polym11050831

**Published:** 2019-05-08

**Authors:** Yanan Wu, Zhengwen Ding, Haohao Ren, Mizhi Ji, Yonggang Yan

**Affiliations:** College of Physical Science and Technology, Sichuan University, Chengdu 610064, China; wuyananchn@126.com (Y.W.); dakuiaa@163.com (Z.D.); renhaohao@scu.edu.cn (H.R.); jimizhichn@163.com (M.J.)

**Keywords:** pearl powder, P/PAA composite, mechanical properties, bioactivity, osteogenic activity

## Abstract

Many studies about fabricating organic-inorganic composite materials have been carried out in order to mimic the natural structure of bone. Pearl, which has a special block-and-mortar hierarchical structure, is a superior bone repair material with high osteogenic activity, but it shows few applications in the clinical bone repair and reconstruction because of its brittle and uneasily shaped properties. In this work, pearl powder (P)/poly (amino acid) (PAA) composites were successfully prepared by a method of in situ melting polycondensation to combine the high osteogenic activity of the pearl and the pliability of the PAA. The mechanical properties, in vitro bioactivity and biocompatibility as well as osteogenic activity of the composites were investigated. The results showed that P/PAA composites have both good mechanical properties and bioactivity. The compressive strength, bending strength and tensile strength of the composites reached a maximum of 161 MPa, 50 MPa and 42 MPa, respectively; in addition, apatite particles successfully deposited on the composites surface after immersion in simulated body fluid (SBF) for 7 days indicated that P/PAA composites showed an enhanced mineralization capacity and bioactivity due to incorporation of pearl powder and PAA. The cell culture results revealed that higher cell proliferation and better adhesion morphology of mouse bone marrow mesenchymal stem cells (MSCs) appeared on the composite surface. Moreover, cells growing on the surface of the composites exhibited higher alkaline phosphatase (ALP) activity, more calcium nodule-formation, and higher expression levels of osteogenic differentiation-related genes (COL 1, RunX2, OCN, and OPN) than cells grown on PAA surface. The P/PAA composites exhibited both superior mechanical properties to the pearl powder, higher bioactivity and osteogenic capability compared with those of PAA.

## 1. Introduction

Bone, a critical tissue responsible for supporting the human body, has been inevitably damaged for a long time of use, and disease attack leads to the study of bone repair materials [1]. Among these bone repair materials, the substitution of load-bearing bone segments is considered to be one of the important and challenging parts in orthopedic surgery [2]. Currently, owing to the scarcity of autologous bone and the immunogenicity of allograft bone, the most commonly used materials in the clinic are almost artificial materials, including biomedical metals, biomedical polymers and composites [3,4,5]. Generally speaking, biomedical metals, such as titanium alloy, have excellent mechanical properties but no suitable modulus to match the human bone tissue and poor fusion with bone [6]. Biomedical polymers have been widely used in clinics due to the selectable component with various functions, such as poly-amino acid (PAA), with the suitable properties of non-toxic, low cost, simple synthetic process, easy processing, strong interaction with filler particles and good biocompatibility [7]. However, the single polymers have seldom been used directly as bone repair materials because of their poor bioactivity and mechanical strength [8]. Most polymers have been modified to possess bioactivity by combining with the bioactive inorganic Ca-contained compounds, such as hydroxyapatite (HA), calcium phosphate, calcium sulphate, which possess excellent bioactivity but have little osteogenic activity [9,10]. Previous works have proved that the presence of bioactive particles not only improves mechanical properties of composites due to stiff particles binding to the soft polymer, but could also endow the polymer with bioactive behaviors [11,12]. The calcium phosphate ceramics-based polymer composites have been widely studied and used for the bone repair materials mainly based on the fact that the human bone is composed of apatite and collagen, a kind of polymer mainly composed of amino acids. Qian et al. prepared a new nano-hydroxyapatite/polyamide 66/glass fiber composite with good biomechanical properties and biocompatibility but no effect on matrix mineralization and osteogenic differentiation of mesenchymal stem cells [13].

One of the interesting things is the main components of the bone tissue of the sea-related animal are calcium carbonated-polymer composites, being different from human bone [14]. For example, pearl, composed of nacre, is produced in an active physiological environment by mollusk. As far as the component is concerned, pearl is made of calcium carbonate (aragonite, CaCO_3_) rather than calcium phosphate (the main component of bone). It is found that pearl contains one or more signal molecules that can activate the osteogenic-related bone marrow cells to form new bone tissue [14,15,16]. Pearl has superior mechanical performance thanks to the organic matrix lying between neighboring tablets and lamellae forming a “brick and mortar” structure whose formation is dictated by the organic matrix [17,18]. Moreover, pearl is a natural carrier of bone growth factors which will behave high osteogenic activity and stimulate osteoblast proliferation [19,20]. Atlan et al.’s study on defects in human alveolar maxillary bone implanted with nacre showed that new bone formation extended deep into the nacre implant in all patients, indicated that nacre powder could promote new bone formation [21]. Furthermore, compared with nacre, pearl contains more organic substances which endow it with high osteogenic activity [16,22]. Shen et al. have made a detailed study of the osteogenic activity of pearl and proved that pearl could stimulate osteoblast proliferation, which proceeded more quickly and smoothly than nacre and HA [16]. Although pearl is a suitable natural substance for bone repair, its applications are limited because of its brittleness and difficulty in molding.

In this study, biomimetic pearl powder (P)/poly-amino acid (PAA) composites were designed as the load-bearing bone repaired materials. The PAA as the matrix provided the required mechanical properties for the composites and solved the problem of pearl powder in molding, and pearl powder as an inorganic additive endowed the composites with osteogenic activity and enhanced the mechanical properties. P/PAA composites were prepared through in situ melting polycondensation, a convenient and effective way to prepare a composite. This study was mainly focused on the preparation and characterization of the P/PAA composites, mechanical properties, evaluation of in vitro bioactivity, degradation, cytocompatibility and osteogenic activity in order to provide a basis for further animal model and physiological function research.

## 2. Materials and Methods

### 2.1. Materials

6-aminocaproic acid, L-hydroxyproline, L-alanine, L-phenylalanine, L-proline and L-lysine (99.0%, biochemical grade) were purchased from Hebei kairuijie amino co., Ltd. (Xintai, China). The pearl powder with 0.5–10 µm diameter was purchased from STS Biotech Co., Ltd. (Wuxi, China). Minimum essential medium eagle-alpha modification (α-MEM), fetal bovine serum (FBS), 100 U/mL penicillin and 100 mg/mL streptomycin sulfate were purchased from Gibco (Grand Island, NY, USA); Cell counting kit-8 (CCK-8) was purchased from Nanjing Keygen Biotech Co., Ltd. (Nanjing, China); Glutaraldehyde (50.0%, AR) and formaldehyde (37.0–40.0%, AR) were purchased from Chengdu Chron Chemicals Co., Ltd. (Chengdu, China); Rhodamine-phalloidin was obtained from Servicebio Co., Ltd. (Wuhan, China), Dexamethasone (98.0%), ascorbic acid (99.0%), β-glycerophosphate sodium (99.0%) and hexadecylpyridinium chloride (99.0–102.0%) were purchased from Sigma-Aldrich Co., Ltd. (Saint Louis, MO, USA); Mouse Alkaline Phosphatase Activity (ALP) Enzyme-linked Immunosorbent Assay (ELISA) Kit was purchased from Shanghai MLBIO Biotechnology Co., Ltd., Alizarin Red S solution was purchased from Cyagen Biosciences Co., Ltd. (Guangzhou, China), and TRIZOL reagent from Ambion (Carlsbad, CA, USA).

### 2.2. Preparation and Characterization of the P/PAA Composites

**Figure 1:**
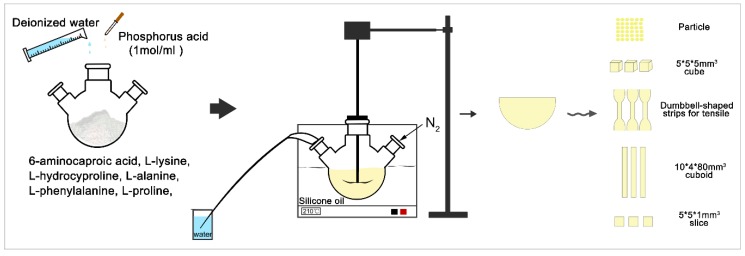
The P/PAA composite fabrication using in situ melting polycondensation methods.

The P/PAA composites were fabricated by a method of in situ melting polycondensation (Figure 1) [23]. First, 105 g 6-aminocaproic acid, 6 g L-hydroxyproline, 5 g L-alanine, 13 g L-phenylalanine, 3 g L-proline, 5 g L-lysine, 50 mL deionized water, and 0.5 mL (1 mol/L) phosphorus acid as the catalyst were added in five three-necked flasks. The mixture was heated to 190 °C for about 2 h using an oil bath until the water was evaporated under the protection of nitrogen. The reaction was then performed at 210 °C for 2 h. Second, 0, 51, 79, 119 g of pearl powder were correspondingly added to the four three-necked flasks, for 30 min at 210 °C. Finally, the mixture was maintained in the three-necked flasks for cooling to room temperature. The obtained composites containing 0, 30, 40, and 50% (mole fraction) of pearl powder were named PAA, 30P/PAA, 40P/PAA, and 50P/PAA composites, respectively. The PAA sample was used as a control.

The crystal phases of the P/PAA composites and PAA were analyzed by X-ray diffraction (XRD; X’ Pert Pro-MPD, Panalytical, Almelo, The Netherlands) using Cu/K_α_ radiation with wavelength of 0.154 nm at 40 kV and 200 Ma over the range of 10–80°. The functional groups of the specimens were analyzed by Fourier transform infrared spectroscopy (FTIR; Nicolet 6700, Thermo scientific, Waltham, MA, USA). The spectral resolution was 4 cm^−1^, ranging from 4000 to 400 cm^−1^. Surface chemical composition of the samples was measured using X-ray photoelectron spectroscopy (XPS; XSAM800, Shimadzu, Kyoto, Japan). The scan spectrum was obtained over a range of 1–1100 eV.

### 2.3. Mechanical Properties

The mechanical properties (including the compressive strength, tensile strength and bending strength) of the composites were tested using a mechanical testing machine (Instron, Norwood, MA, USA) equipped with a 10 kN load cell. The mechanical testing specimens were moulded into the size of 5 × 5 × 5 mm^3^ for compressive strength, 80 × 10 × 4 mm^3^ for bending strength, 2 mm thick and 4 mm wide dumbbell-shaped strips for tensile strength. The samples were tested with a controlled speed of 5 mm/min at room temperature, and the load was applied until the specimens were compressed to either approximately 60% of their original height or until the sample collapsed. Three replicates were carried out and each value obtained represented the average of samples. The results were expressed as mean ± standard deviation (SD).

The densities of the samples were measured by the Archimedes method using a pycnometer and a glass bottle of known volume with a capillary tube at the top as a container. The liquid medium was distilled water for all materials.

### 2.4. In Vitro Degradation

The samples, about 0.3 g each, were soaked in individual centrifuge tubes with 9 mL phosphate buffer solution (PBS) in a shaking water bath (SHA-CA, Changzhou Putian Instrument Manufacturing Co., Ltd., Changzhou, China) at 37 °C at 70 rpm/min. The initial pH of PBS was 7.40. The medium was replaced by fresh PBS every week and the pH value of each sample was detected using a pH meter (PHS-3C, INESA, Shanghai, China) after soaking for different periods (1, 4, 7, 14, 21, 28 days). Then, the samples were taken out at the different incubation periods, and the samples were dried at 80 °C for 8 h until the weight of samples were constant. For the original samples, not soaking into PBS, also were dried at the 80 °C as the control. The weight loss was calculated as follows:

(1) $$\mathrm{Weight}\;\mathrm{Loss}\left(\%\right)=\frac{m_{i}-m_{j}}{m_{i}}\;\times \;100\%$$

Here, $m_{i}$ denoted the original dried mass of sample, $m_{j}$ denoted the residual dried mass after soaking in solution for different periods.

### 2.5. Apatite Mineralization

In vitro bioactivity of an implanted material is generally evaluated by the formation of a bone-like apatite layer on its surface in the simulated body fluid (SBF). The P/PAA composites and PAA cut into the size of 5 × 5 × 5 mm^3^ were rinsed by ultrasonic in deionized water three times and dried overnight, and then soaked in SBF at 37 °C for 7 days. The simulated body fluid was prepared according to the method described in reference [24] and the SBF was renewed every 2 days. After predetermining intervals, the composites were removed from SBF, washed with deionized water, dried overnight, sputter-coated with gold, and analyzed by scanning electron microscopy (SEM; JSM-5600LV, JEOL, Tokyo, Japan) and energy dispersive spectrometry (EDS). Additionally, the concentrations of calcium (Ca) ions in the soaked SBF at 1, 3, 5, and 7 days were measured by inductively coupled plasma atomic emission spectroscopy (ICP-MS, Thermo Jarrell Ash Co., Waltham, MA, USA).

### 2.6. Cell Cytotoxicity and Proliferation

Mouse bone marrow mesenchymal stem cells (MSCs) (ATCC; Chinese Academy of Sciences, Shanghai, China) were incubated in a growth medium containing α-MEM medium with 10% FBS, 1% penicillin (100 U/mL) and streptomycin (100 mg/mL) at 37 °C in a humidified atmosphere containing 5% CO_2_. The culture medium was changed every 2 days. A cell counting kit-8 (CCK-8) assay was applied to evaluate the cell cytotoxicity and proliferation of the P/PAA composites and PAA. The samples were rinsed by ultrasonic in deionized water and soaked in 75% ethanol aqueous solution for at least 1 day, then were cut into the size of 5 × 5 × 1 mm^3^ and placed in a 48-well plate. Cultured MSCs were seeded in each well at a density of 1 × 10^4^/mL and incubated at 37 °C in a humidified atmosphere containing 5% CO_2_. The growth medium was changed every 2 days. After incubating for a certain time, CCK-8 was mixed with α-MEM at a ratio of 1:10, and the old medium was removed. Then, 250 µL of the mixed solution was added to each well and the plates were incubated at 37 °C in a humidified atmosphere containing 5% CO_2_ for 3 h. Then, 100 µL solution was taken out from each well and added into the 96-well plate. Then, the absorbance of the solution at 450 nm was measured using a microplate reader (Multiskan FC, Thermo Fisher Scientific, Shanghai, China).

### 2.7. Cellular Morphology and Cytoskeletal Observation

The cellular morphology and cytoskeleton on the samples were observed via SEM and confocal laser scanning microscopy (CLSM).

To prepare the cell samples for SEM, the MSCs were seeded and incubated on samples (5 × 5 × 1 mm^3^) placed in a 12-well plate for 3 days. On day 3, the samples were washed with phosphate buffered saline (PBS), fixed with 2.5% glutaraldehyde for 4 h in 4 °C and washed again with PBS. Then, the cells were dehydrated with gradient ethanol at volume fractions of 20%, 30%, 40%, 50%, 70%, 90%, and 100% (15 min for each gradient). Finally, the samples were dried under vacuum, gold coated, and examined with SEM.

To prepare the cell samples for CLSM, the MSCs were also seeded and incubated on samples (5 × 5 × 1 mm^3^) placed in a glass bottom cell culture dish (Ф 15 mm). After culturing on the samples for 3 days, the cells were gently washed three times with PBS, fixed with 4% formaldehyde for 15 min, and permeabilized with 0.5% Triton X-100 in PBS for 10 min. Then, the cells were stained with rhodamine-phalloidin for 30 min and washed three times with PBS (5 min for each washing). The filamentous actins of the cell cytoskeleton were visualized using a CLSM (TCS SP2, Leica, Heidelberg, Germany).

### 2.8. Alkaline Phosphatase Activity (ALP)

The MSCs were seeded onto the PAA and P/PAA composites in a 12-well plate at a density of 5 × 10^4^/mL in the growth medium and incubated for 24 h, and then the growth medium was changed to the osteogenic medium containing α-MEM medium with 10% FBS, 1% penicillin (100 U/mL) and streptomycin (100 mg/mL), 100 nM dexamethasone, 50 µg/mL ascorbic acid, and 10 mM β-glycerophosphate sodium. The osteogenic medium was changed every 2 days. After 4, 7, and 10 days of culture, the alkaline phosphatase activity was measured with a Mouse Alkaline Phosphatase Activity (ALP) Enzyme-linked Immunosorbent Assay (ELISA) Kit. All procedures were done according to the manufacturer’s protocols.

### 2.9. Calcium Deposition Assay and Methylene Blue Staining

Alizarin red staining was used to analyze calcium nodule formation (mineralization). The MSCs were grown on the samples for 21 days in osteogenic medium, as described previously. The cells on the samples were fixed in 4% formaldehyde for 15 min, and rinsed with distilled water. Then, 1 mL of pH 4.2 Alizarin Red S solution was added to cover cell surface for 10 min, followed by washing thoroughly with distilled water. The calcium deposits exhibited as orange red sediments on the cell surface and were recorded microscopically.

The MSCs were cultured for 7 days and fixed as described above. Then, 1 mL of 1% methylene blue solution was added to cover cell surface for 1 min, followed by washing thoroughly with distilled water. Then, the morphology of cells cultured in osteogenic medium for 7 days was observed by inverted phase contract microscope (ECLIPSE Ti, Nikon, Tokyo, Japan).

### 2.10. Real-time quantitative PCR (PT-qRCR) analysis

The osteogenic differentiation-related genes, i.e., osteopontin (OPN), osteocalcin (OCN), Runt-related transcription factor 2 (RunX2), collagen type I (COL I) were analyzed via a real-time fluorescent quantitative polymerase chain reaction (RT-qPCR, Stepone plus, ABI), using β-ACTIN as the housekeeping gene. The MSCs were seeded and cultured in osteogenic medium, as described previously. The samples were harvested on the seventh day. The sequences of the forward and reverse primes are shown in Table 1. Total RNA of the MSCs grown on the samples was isolated using a TRIZOL reagent. Reverse transcription was performed following the protocol of the RevertAid First Strand cDNA Synthesis Kit (Fermentas, Thermo Scientific Molecular Biology, Pittsburgh, PA, USA). RT-qPCR was performed using the FastStart Universal SYBR Green Master (Rox) (Roche, Basel, Switzerland).

### 2.11. Statistical Analyses

Triplicate experiments were performed for each sample, and data were expressed as the mean ± standard deviation. The statistical analysis was carried out through one-way analysis of variance (ANOVA). The differences were considered statistically significant at *p* < 0.05, highly significant at *p* < 0.01.

## 3. Results

### 3.1. XRD and FTIR Analysis of the P/PAA Composites

**Figure 2:**
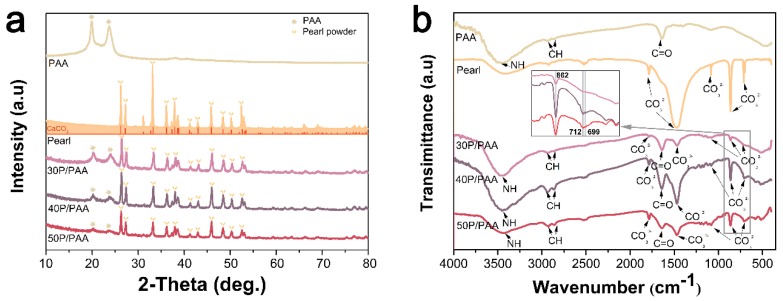
XRD (**a**) and FTIR (**b**) of the pearl, PAA, and the P/PAA composites.

Figure 2a presents the XRD pattern of the pearl, PAA, and the P/PAA composites. It could be seen that the diffraction peaks of pearl powder were mostly consistent with spectrogram of the calcium carbonate (aragonite) (JCPDS, NO.41–1475). The diffraction peaks of the composites at approximately 2θ = 26°, 27°, 33°, 36°, 38°, 41°, 43°, 46°, 48°, 50°, and 52° were attributed to the characteristic peaks of pearl. The two peaks at approximately 2θ = 20° and 24° belonged to the characteristic peaks of PAA. The diffraction peaks of PAA became weaker gradually with the increase of the pearl powder content. The results indicated that the composites contained both pearl and PAA.

Figure 2b shows the FTIR pattern of the pearl, PAA, and the P/PAA composites. The absorption peak at 3440 cm^−1^ represented the stretching vibration of N–H of PAA. The peaks at 2937 cm^−1^, 2851 cm^−1^ were attributed to the characteristic peaks of methyl and methylene groups. The band around 1543 cm^−1^ represented the stretching vibrations of carbon-nitrogen. The peak at 1638 cm^−1^ belonged to the stretching vibrations of carbonyl groups in PAA, caused by the interaction between -NH_2_ group and -COOH group. The peaks at 1787 cm^−1^ and 1470 cm^−1^ were assigned to the antisymmetric stretching vibration peaks of CO_3_^2−^ of pearl, the peaks at 1083 cm^−1^ were related to the symmetrical stretching vibration peaks of CO_3_^2−^ of pearl, the peaks at 862 cm^−1^ belonged to the out-of-plane bending vibration peaks of CO_3_^2−^ of pearl, and the peaks at 712 cm^−1^ and 699 cm^−1^ were assigned to the in-plane bending vibration peaks of CO_3_^2−^ of pearl [25]. The above peaks were present in the composites. The aragonite specific double band of CaCO_3_ in pearl at 712 cm^−1^ and 699 cm^−1^ were present in the composites, which could confirm that the structure of pearl remained unchanged [26,27], but some of the characteristic absorption peaks had slight shifts in the composites which might be caused from the interface interaction between the inorganic and polymer matrix [28]. The results also indicated that the composites contained both pearl and PAA.

### 3.2. XPS Analysis of the P/PAA Composites

**Figure 3:**
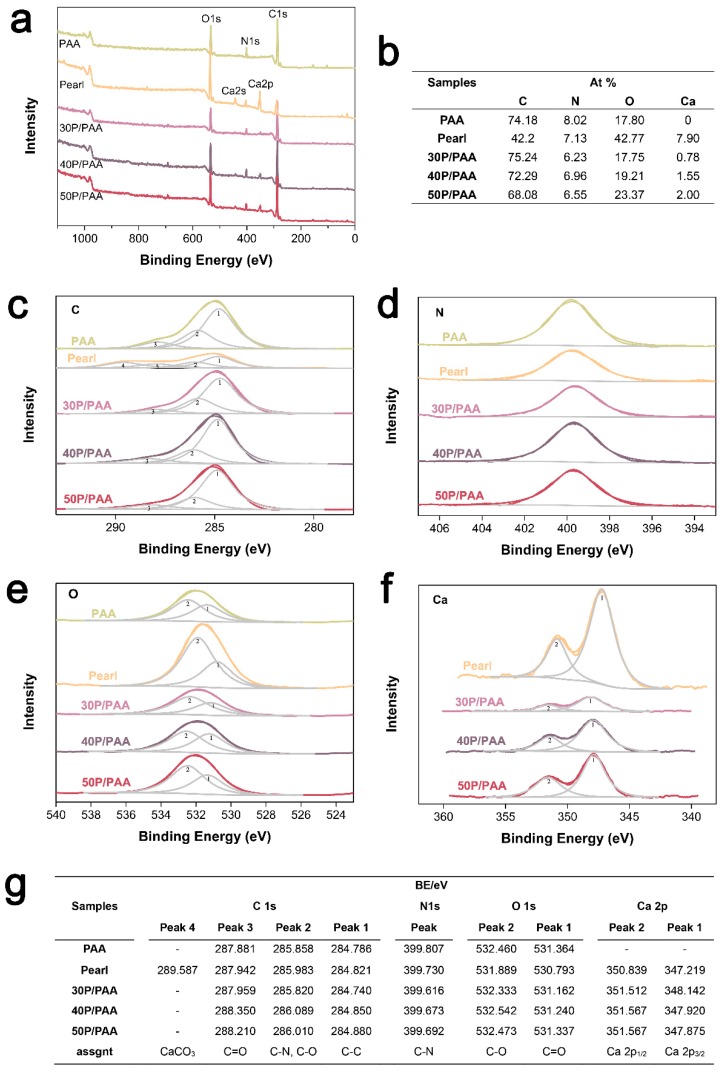
XPS spectra of the pearl, PAA, and the P/PAA composites. (**a**) XPS wide spectra, (**b**) atomic concentration (at%) of surface elements as detected by XPS, high-resolution XPS spectra of (**c**) C 1s, (**d**) N 1s, (**e**) O 1s, and (**f**) Ca 2p, (**g**) The binding energy (BE) for C 1s, N 1s, O 1s, and Ca 2p in different states in samples.

Figure 3 shows the XPS data of the pearl, PAA, and the P/PAA composites. XPS wide scan (Figure 3a) identified carbon, nitrogen, oxygen, calcium (from pearl powder) as the major constituents of the P/PAA composites. The relative atomic concentration for C and N of the composites slightly decreased compared with PAA, whereas O and Ca increased. From high-resolution XPS spectra of C 1s, it could be seen that Peak 4 (289.587 eV, belonged of CaCO_3_) of pearl powder disappeared in the composites [29]. The spectra shape of N 1s, O 1s and Ca 2p of the composites had little differences with PAA. As shown in Figure 3g, the binding energies of Peak 3 (287.959–288.350 eV for C=O), Peak 2 (285.820–286.089 eV for C-N and C-O), Peak 1 (284.740–284.880 eV for C–C) of the composites C 1s were all higher than those of PAA [29], and the binding energies of the composites Ca 2p were also higher than those of pearl powder, while the binding energies of the composites N 1s (399.616–399.692 eV for C–N) were less than those of PAA [29]. The binding energies of the composites O 1s (C–O and C=O) were higher than those of pearl powder but slightly less than PAA. The changed binding energies indicated a possible interaction between PAA and pearl powder.

### 3.3. Mechanical Properties

Table 2 lists the mechanical properties of the P/PAA composites with different pearl powder contents. It could be seen that the addition of pearl powder enhanced the mechanical properties of the PAA. The composite with 50 wt % pearl powder resulted in the highest compressive strength of 161 MPa, the highest bending strength of 50 MPa and the highest tensile strength of 42 MPa. Moreover, the density of the PAA was also enhanced by the introduction of pearl powder.

### 3.4. Weight Loss and pH Value

**Figure 4:**
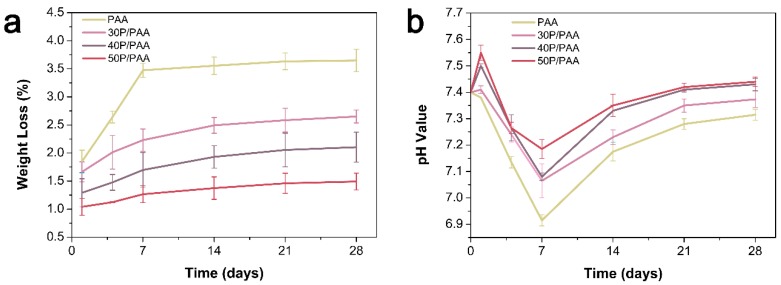
Weight loss (**a**) of the PAA and composites and pH variation (**b**) of the medium after soaking in PBS for different times.

The weight loss of the PAA and P/PAA composites which were immersed in PBS for certain intervals is shown in Figure 4a. The weight loss of PAA increased in the first two weeks, then showed slight change in the next two weeks. The total weight loss of the PAA, 30P/PAA, 40P/PAA and 50P/PAA was 3.64%, 2.60%, 2.07% and 1.46%, respectively, after 28 days soaking.

The pH value of the PAA and P/PAA composites after immersing in PBS for certain intervals is shown in Figure 4b. The pH value of PBS-soaked PAA decreased from initial 7.40 to 6.92 during the first week, followed by a growth until reached up to 7.32 in the twenty-eight day. Meanwhile, the pH value of PBS-soaked the composites slightly increased in the first day and decreased to the minimum of 7.06 on the seventh day, then followed by a rebound. After 28 days, the pH value of the 30P/PAA, 40P/PAA, 50P/PAA composites soaked in PBS was 7.37, 7.43, 7.44, respectively.

### 3.5. Apatite Mineralization

**Figure 5:**
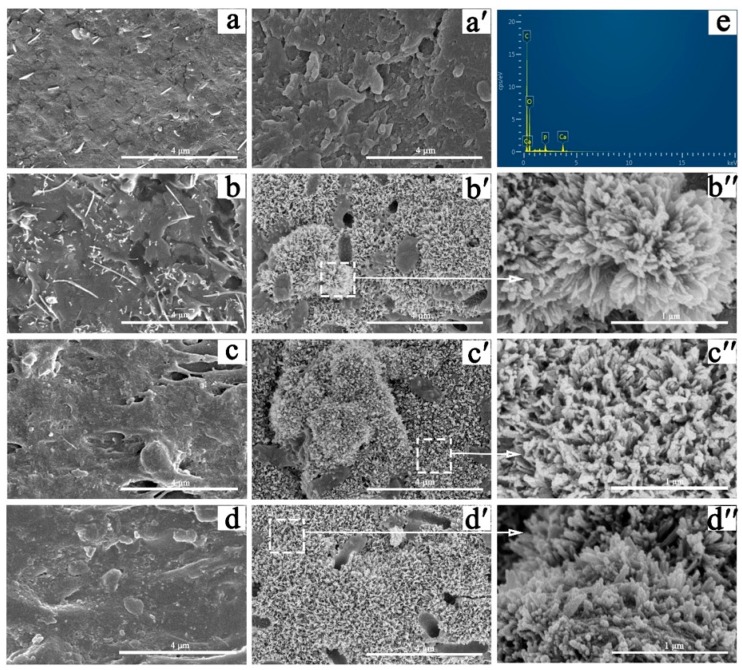
Apatite-formation on the PAA and composites after immersion in SBF for 7 days. The SEM photographs of the PAA and the composites before soaking in SBF: (**a**) PAA, (**b**) 30P/PAA, (**c**) 40P/PAA, (**d**) 50P/PAA (scale bar = 4 µm); and after soaking in SBF for 7 days: (**a′**) PAA, (**b′**) 30P/PAA, (**c**′) 40P/PAA, (**d**′) 50P/PAA (scale bar = 4 µm), (**b′′**) 30P/PAA (scale bar = 1 µm); (**c**′′) 40P/PAA (scale bar = 1 µm); (**d**′′) 50P/PAA (scale bar = 1 µm); (**e**) EDS spectra of the 50P/PAA composite after SBF soaking 7 days.

**Figure 6:**
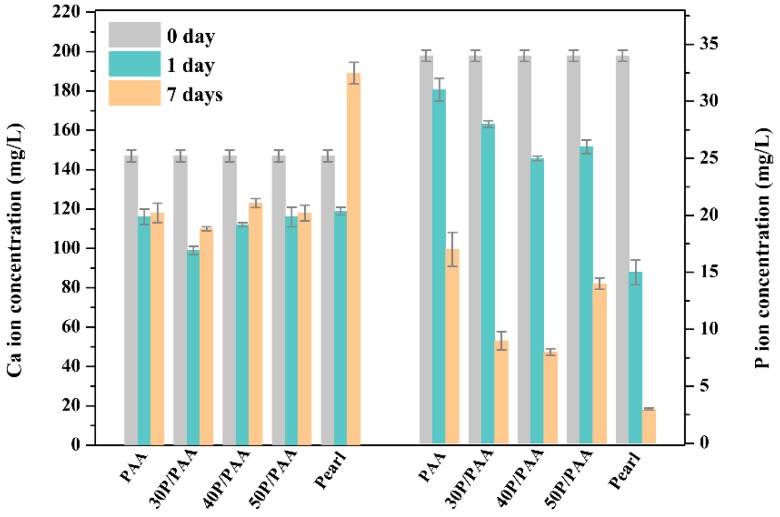
Variation of the Ca ion (left) and P ion (right) concentrations of SBF after the samples soaked for 0, 1, and 7 days.

Figure 5 shows SEM images of the surface morphology of the PAA and P/PAA composites before and after SBF soaking for a week. Comparison of initial surface (Figure 5b–d) and post-soaking (Figure 5b′–d′) of the P/PAA composites revealed that a pronounced change had occurred; all the P/PAA surfaces were covered by a layer of newly formed particles. After enlarging the deposited particles (Figure 5b′′–d′′), it could be seen that a large particle was composed of some irregular worm-like particles. However, comparing the surface morphology of PAA before (Figure 5a) and after (Figure 5a’) soaking, there was no newly formed substance on PAA after SBF soaking for a week. The corresponding composition analysis of the deposited particles in 50P/PAA composite had also been done by EDS, as shown in Figure 5e. Ca and P peaks appeared on the surface of the composite after SBF soaking for a week. It could be inferred that the deposited particles were apatite particles. Figure 6 shows the variations of Ca and P ions concentrations in SBF after incubating with the PAA, pearl powder and the composites after various time periods. The results indicated that the Ca ion concentration in SBF after incubating for one day with PAA and the composites decreased, then slight increased on the seventh day, while the Ca ion concentration of pearl-soaked SBF also decreased after one day, then increased rapidly. The P ion concentration in SBF after incubating with PAA, pearl powder and the composites decreased gradually from day 0 to day 7 and the lowest P ion concentration was found in pearl-soaked SBF at day 7.

### 3.6. Cell Proliferation

**Figure 7:**
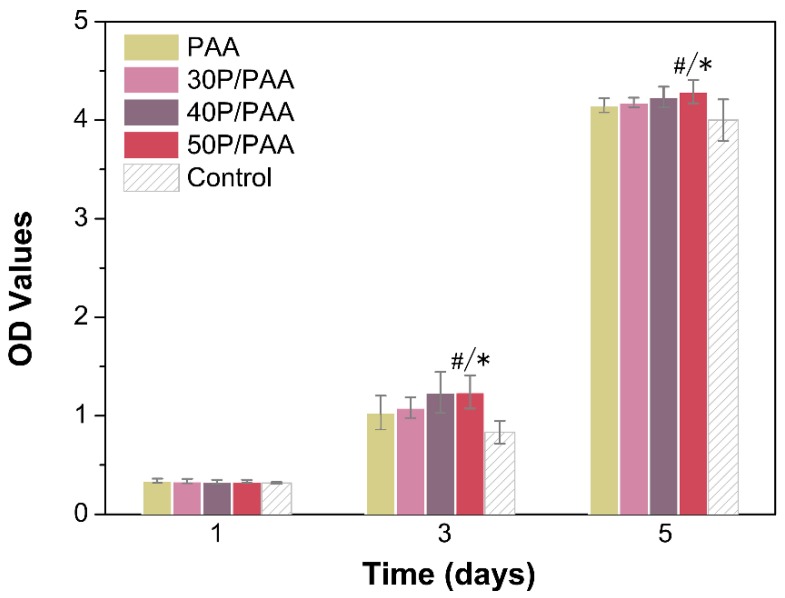
Cell proliferation on PAA and the composites for 1, 3 and 5 days based on a cell counting kit-8 assay. * Significant difference compared with control group (*p* < 0.05); # significant difference compared with PAA group (*p* < 0.05).

To evaluate cell proliferation on PAA and the P/PAA composites at 1, 3, and 5 days, the CCK8 assay was carried out. The obtained results were displayed in a bar graph of OD-culture time (Figure 7). In Figure 7, we could see that as the incubation time increased, MSCs proliferation increased significantly in all groups. The cell proliferation on the PAA and P/PAA composites had no significant difference after cell culturing for 1 day. After cell culture for 3 and 5 days, more cells were observed to attach on the 50P/PAA group than the control group (* *p* < 0.05) and PAA group (^#^ *p* < 0.05) after 5 days. These results indicated that the P/PAA composites were not cytotoxic and pearl powder could provide a good culture condition for cells.

### 3.7. Cellular Morphology and Cytoskeletal Observation

**Figure 8:**
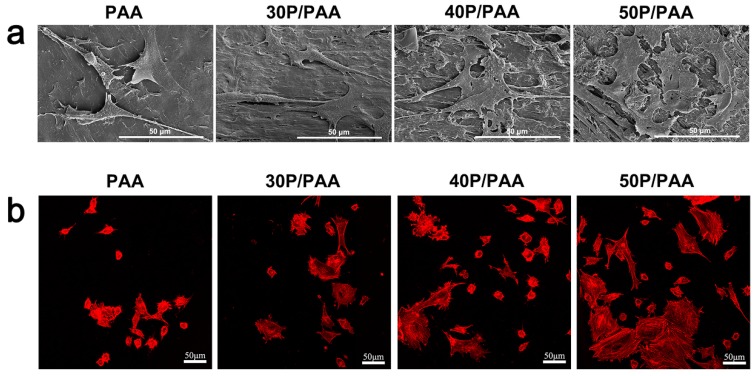
(**a**) SEM images of the cell morphology and spreading on PAA and the composites after 3 days of culture. The scale bar is 50 µm; (**b**) CLSM images of the cytoskeleton of MSCs grown on PAA and the composites for 3 days (scale bar = 50 µm).

To observe the attachment and morphology of MSCs on the composites, SEM images were taken three days after seeding (Figure 8a). After 3 days of incubation, cells attached on the samples surface and extended properly. It could be seen that MSCs on the composite groups exhibited elongating flat morphologies, anchoring onto the composites through numerous filopodia and lamellipodia, and cell-cell interactions were observed as well. A higher level of cell spreading into a typically polygonal morphology could be observed on the composites. This evidence showed that the composites had good cytocompatibility, promoting the attachment of cells and their growth on the surfaces of the composites.

The cytoskeleton of the MSCs seeded on different samples was observed by confocal laser scanning microscopy after culturing for 3 days. In Figure 8b, cells grown on the composites were polygonal and clustered, whereas cells grown on PAA were dispersive, and with the increase of pearl powder, we could see more actin filaments linking adjacent cells on the composites than on PAA. The results indicated that the existence of pearl powder was beneficial for cell proliferation and adhesion.

### 3.8. Alkaline Phosphatase (ALP) Activity

**Figure 9:**
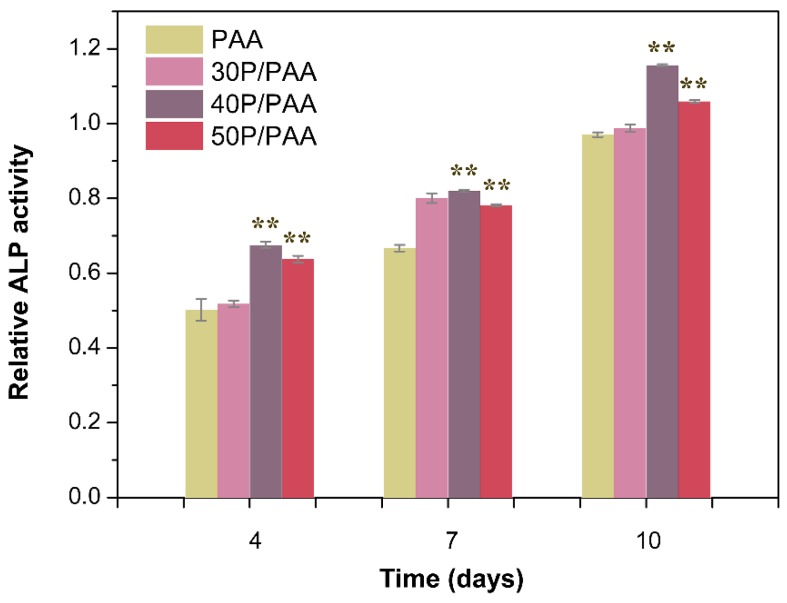
ALP activity of cells grown on PAA and the composites after 4, 7, and 10 days. ** Significant difference compared with PAA (*p* < 0.01).

The ALP activity was applied to evaluate the potential osteoblastic differentiation of MSCs on the composites, shown in Figure 9. The ALP activity on the P/PAA composites was significantly higher than that on PAA (** *p* < 0.01). Such an improvement could be associated with the incorporation of the pearl powder phase into the PAA matrix, enhancing osteoblastic differentiation of the cells.

### 3.9. Calcium Deposition Assay

**Figure 10:**
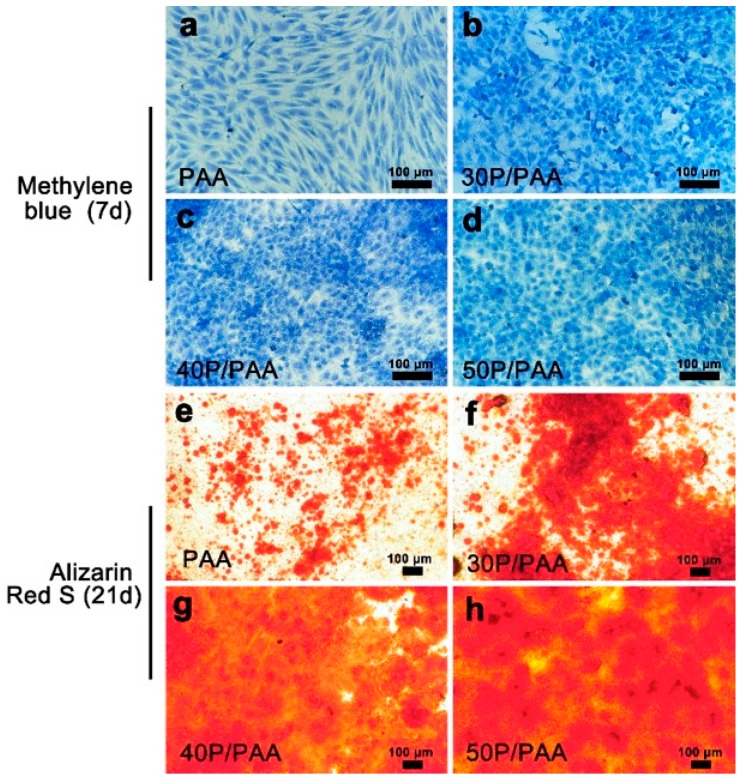
Methylene blue solution staining on PAA and the composites after 7 days: (**a**) PAA, (**b**) 30P/PAA, (**c**) 40P/PAA, (**d**) 50P/PAA; Alizarin red staining on PAA and the composites after 21 days: (**e**) PAA, (**f**) 30P/PAA, (**g**) 40P/PAA, (**h**) 50P/PAA. The scale bar is 100 µm.

After 7 days of culturing in osteogenic medium, we observed the cell morphology by methylene blue staining and found the cell morphology was normal (Figure 10a–d). The cells in the PAA group were spindle shaped. The morphological change from spindle to polygon shape was observed by day 7 with the increasing concentration of pearl powder. Specially, in the presence of pearl powder, MSCs became more round in groups of the composites as compared to group PAA, indicating their tailored cell fate by the role of pearl powder in the composites. Moreover, under the influence of pearl powder, cells were not much different in number among the groups of the composites, but slightly higher in number as compared to the PAA group. After 21 days culture, we assessed the calcium mineralization using alizarin red staining that is used to prove the calcium deposition and nodules. Alizarin red S can selectively associate with calcium ions and produce orange red sediments. We observed the mineralized calcium deposition and nodules in all groups in Figure 10e–h, which indicated MSCs were differentiated into osteoblast lineage. Compared with the PAA group, a large amount of bone like nodules are found in P/PAA composite groups.

### 3.10. Real-Time Quantitative PCR

**Figure 11:**
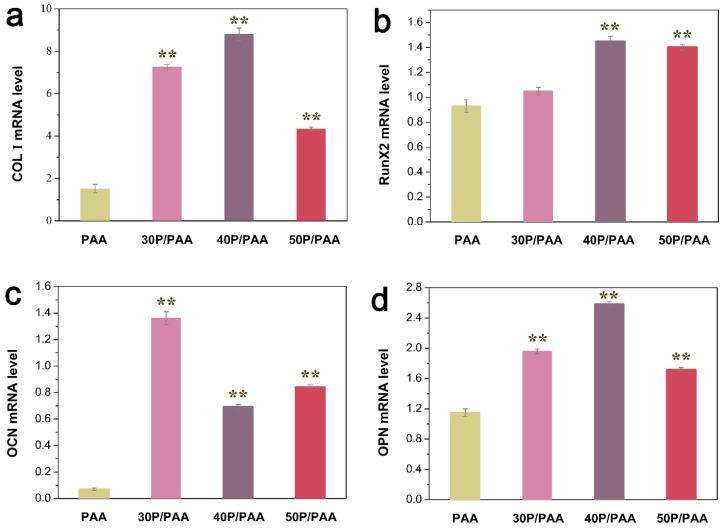
Relative mRNA expression of osteogenic differentiation-related genes in MSCs grown on PAA and the composites on day 7 measured by real-time PCR: (**a**) COL I, (**b**) RunX2, (**c**) OCN, (**d**) OPN. The mRNA levels of above using ACTIN as a reference. ** Significant difference compared with the PAA group (*p* < 0.01).

The gene expression levels of osteogenesis-related factors, collagen type I (COL I), Runt-related transcription factor 2 (RunX2), osteocalcin (OCN), and osteopontin (OPN) required for pre-osteoblast differentiation into mature osteoblasts were detected to investigate the osteogenic potential of the composites (Figure 11). After induction for 7 days, the mRNA expression levels of COL I (Figure 11a), RunX2 (Figure 11b), OCN (Figure 11c) and OPN (Figure 11d) in the composites were significantly higher than those in the PAA group (** *p* < 0.01). The result demonstrated that the addition of pearl powder could enhance osteogenic activity of MSCs.

## 4. Discussion

The present study investigated a novel P/PAA composite combining the bioactivity and osteogenic activity of pearl powder with the elasticity and plasticity of the PAA [16]. There are a lot of factors influencing the chemicophysical and biological properties of the composites, especially the interface combination between the different components of the composites [6]. Combining the XRD, IR and XPS analysis in Figure 2 and Figure 3, it could be inferred that some strong bonding interface and interactions are present between PAA and pearl in the composites. The XRD data showed that the diffraction pattern of pearl powder in the composites is mostly identical to original pearl powder, indicating the pearl powder keeps the original crystal structure of pearl [26]. This can be further confirmed by the presence of the aragonite specific double bands of CaCO_3_ in pearl at 712 cm^−1^ and 699 cm^−1^ in the composites. The decreased crystallinity of PAA in the composites was due to pearl powder intercalated amid the PAA chains which reduced the molecular chain regularity of the PAA. From the FTIR spectra (Figure 2b), there were slight shifts of characteristic absorption peaks in the composites compared with original pearl and PAA. These slight shifts confirmed that there was a certain interface interaction between inorganic (pearl) and polymer matrix (PAA). This was in accordance with the XPS data (Figure 3). The binding energies of Ca 2p for the composites were higher than those of pearl powder, which may be due to the –COO^−^ group of PAA bonding with the Ca^2+^ of pearl powder [28]. The binding energy of –COO^−^–Ca^2+^ bond was stronger than that of inorganic Ca^2+^ –CO_3_^2−^ bond because the organic bond had ionic and covalent properties [30]. The O 1s binding energies of the P/PAA composites were lower than PAA but higher than pearl powder because when Ca^2+^ was coordinated with –COO^−^, the bond C=O was weakened and the bond Ca-O was strengthened. The C 1s Peak 4 (289.587 eV, belonged to CaCO_3_) of pearl powder disappeared in the composites, indicating that there might be some bonds between CO_3_^2−^ of pearl and –C=O and -NH- group of PAA [29]. Thus, it could be concluded that some strong bonding interface and interactions are present between PAA and pearl interface in the composites, which would show positive effects on the mechanical properties of the composites [28].

Biomechanical behaviors are the other key factor which determined the clinical application of the biomaterials [31]. In the present research, the compressive strength and the modulus could be adjusted by changing the pearl powder content in the composites, showed in Table 2. The highest compressive, bending and tensile strengths were 161 MPa, 50 MPa and 42 MPa, respectively, with 50 wt % pearl powder in the composite, which are strong enough for a bone implant [2]. Increased mechanical properties of the composites might be due to the filler reinforcing effect of pearl powder (compared with slightly reduced crystallinity, it played a leading role) and strong interfacial interaction facilitated the effective transmission of loads. This was consistent with some studies that improved the mechanical properties of polymers by adding inorganic materials [32]. The other factor associated the biomechanical behaviors of the composites is the stability of the composites with lower degradation in the host for long-term load bearing [33]. The degradation and weight loss of the P/PAA with different contents of pearl powder in the PBS were 3.64%, 2.60%, 2.07% and 1.46%, respectively, keeping relatively stable after 28 days soaking. This showed that the degraded composites could still remain relatively stable [34]. The degradation of the P/PAA composites can be divided into three parts: the dissolution of small molecules and oligomers in the composites, the hydrolysis of the PAA and the dissolution of pearl powder. The hydrolysis of the PAA is mainly due to the hydrolysis of amide bond (–CO–NH–), and the slightly acidic environment could accelerate the hydrolysis of PAA [35,36]. The formation of apatite on the surface of the composites might be the one reason for the reduced degradation. The micro-degradation may supply ion changes between tissue and materials, promoting the tissue and materials merging together [37].

The bioactivity of the artificial biomaterials is one of the most important factors to be considered as the clinical implantation [31]. The formation of apatite is one of the bioactivity markers and is regarded as the essential requirement for bone-implanted materials [24]. Ni and Ratner reported that apatite could form on nacre surfaces in a phosphate-buffered solution [38]. During the composite soaking in SBF, the Ca ion will be released from the pearl of the composites due to its dissolution. The Ca ion concentration in SBF after incubating with pearl powder or the composites decreased rapidly after 1 day and increased slightly at 7 days (Figure 6). This is attributed to the deposition of the Ca ion from SBF more quickly than its dissolution from pearl after 1 day, and later the accelerating dissolution of pearl powder caused the concentration to rise slightly. The dissolution and deposition of the Ca ion provided favorable conditions for the formation of apatite. Throughout the whole incubation process in the SBF in 7 days, the apatite formed on the P/PAA composite surface. The organic matrix of pearl in the composites precisely controlled the formation process of pearl based on a similar biomineralization mechanism [39,40]. And the organic matrix would play an important role in the formation of apatite on the composites’ surface, acting as some kind of catalyst to promote the precipitation of apatite [16]. These organic matrix could adsorb, bind Ca^2+^ and form Ca^2+^ aggregates at some sites. Then, the accumulation of Ca^2+^ attracted PO_4_^3−^ from SBF and then bound this and deposited it on the composites’ surface, which triggered the formation of apatite nuclei. The apatite nuclei continued to grow by consuming the Ca and P ions in the surrounding fluid to form the apatite layer. The whole process followed a dissolution-binding-precipitation mechanism [16]. In short, compared with PAA, the mineralization ability of the composites was increased with the addition of pearl powder.

Biocompatibility is a critical element in the evaluation of a biomaterial which determines the final destination of the biomaterials [5,41]. There are various factors influencing the biocompatibility, such as pH and the product of degradation. As for the pH shown in Figure 4b, the pH value of the composites ranges from 7.06 to 7.50, suitable for cell survival [42]. The cell culture results showed (Figure 7) that the addition of pearl powder into PAA could slightly promote the proliferation of MSCs. The cells on the composites’ surface exhibited elongating flat morphologies, anchoring onto the composites through numerous filopodia and lamellipodia and a higher level of cell spreading into a typically polygonal morphology. There were more actin filaments linking adjacent cells in the cytoskeleton of the MSCs (Figure 8), attributed to the addition of pearl powder to exert a positive effect on cell attachment and spreading. Furthermore, previous studies have also shown that pearl plays an important role in stimulating osteoblast proliferation [16].

**Figure 12:**
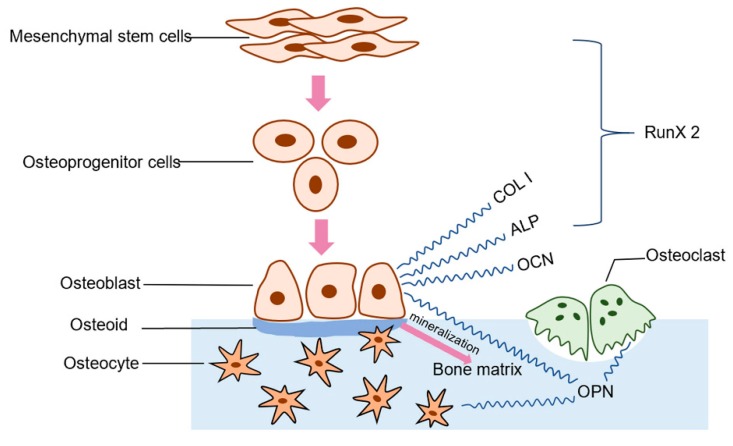
The process of bone formation.

In general, bone formation involves finely orchestrated cellular, molecular events (Figure 12). The osteoblasts originate from the bone marrow mesenchymal stem cells, and after a step by step differentiation program, give rise to mature osteoblasts. The mature osteoblasts first activity is to build the bone, then form osteocytes and bone matrix [43]. Therefore, the ability of osteogenic differentiation and osteogenic activity of biomaterials, which could promote new bone formation, is also crucial.

In the present study, the differentiation of MSCs into osteoblastic phenotype was determined quantitatively using the ALP activity assay, calcium deposition and qRT-PCR test. ALP activity and the expressions of osteogenic differentiation-related genes (Run X 2, COL I, OCN, and OPN) of the cells on the P/PAA composites were significantly higher than those on PAA. This indicated that the composites could promote the osteoblastic differentiation of MSCs, attributed to the addition of pearl powder with high osteogenic activity [44]. Furthermore, the apatite that formed on the composites’ surface also could facilitate differentiation of osteoblasts [45]. In addition, the formation of calcium nodules could indicate that the incorporation of pearl powder promoted the expression of osteogenesis-related genes which controlled the mineralization of osteoid. From the above, the P/PAA composites facilitated the proliferation, attachment, and differentiation of MSCs, which might be a promising bone repair material.

## 5. Conclusions

In this work, the P/PAA composites were fabricated by a method of in situ melting polycondensation. The incorporation of pearl powder into PAA increased the mechanical properties; compressive, bending, tensile strength and bending modulus of the composites, and the 50P/PAA composite showed the optimally mechanical properties. Mineralization ability of these composites was greatly enhanced due to natural pearl powder, which acts as a crystal nucleus for apatite particle deposition. The results revealed that higher cell proliferation and better adhesion morphology appeared on the P/PAA composites. In addition, the introduction of pearl powder enhanced the osteogenic differentiation and activity of the composites compared with PAA. Above all, it could be believed that the P/PAA composites would be promising candidates as a novel osteoconductive composite material for loading-bear bone repair.

## Figures and Tables

**Table 1:** Primers sequence used for RT-qPCR.

Gene	Forward Primer	Reverse Primer
**β-ACTIN**	5-GTGACGTTGACATCCGTAAAGA-3	5-GTAACAGTCCGCCTAGAAGCAC-3
**OPN**	5-TTTCACTCCAATCGTCCCTACA-3	5-CTGCCCTTTCCGTTGTTGTC-3
**OCN**	5-TTTCTGCTCACTCTGCTGACC-3	5-CAGCACAACTCCTTCCTACCA-3
**RunX2**	5-AGCGGACGAGGCAAGAGTTT-3	5-AGGCGGGACACCTACTCTCATA-3
**COL I**	5-AAGAAGCACGTCTGGTTTGGAG-3	5-GGTCCATGTAGGCTACGCTGTT-3

**Table 2:** Mechanical properties of P/PAA composites with different pearl powder contents. ** Significant difference compared with PAA (*p* < 0.01).

Sample	Compressive Strength (MPa)	Bending Strength (MPa)	Tensile Strength (MPa)	Density (g/cm^3^)
**PAA**	100 ± 5	23 ± 1	27 ± 1	1.102 ± 0.005
**30P/PAA**	133 ± 12 **	36 ± 4 **	34 ± 1 ^**^	1.347 ± 0.007 **
**40P/PAA**	139 ± 8 **	47 ± 2 **	38 ± 1 **	1.489 ± 0.003 **
**50P/PAA**	161 ± 10 **	50 ± 2 **	42 ± 1 **	1.608 ± 0.001 **
